# Randomized controlled trial of piperacillin-tazobactam, cefepime and ertapenem for the treatment of urinary tract infection caused by extended-spectrum beta-lactamase-producing *Escherichia coli*

**DOI:** 10.1186/s12879-017-2502-x

**Published:** 2017-06-07

**Authors:** Yu Bin Seo, Jacob Lee, Young Keun Kim, Seung Soon Lee, Jeong-a Lee, Hyo Youl Kim, Young Uh, Han-Sung Kim, Wonkeun Song

**Affiliations:** 1 Division of Infectious Diseases, Department of Internal Medicine, Kangnam Sacred Heart Hospital, Hallym University College of Medicine, Seoul, Republic of Korea; 2 Department of Internal Medicine, Yonsei University Wonju College of Medicine, Wonju, Republic of Korea; 3 Division of Infectious Diseases, Department of Internal Medicine, Hallym University Sacred Heart Hospital, Hallym University College of Medicine, Seoul, Republic of Korea; 4 Department of Laboratory Medicine, Yonsei University Wonju College of Medicine, Wonju, Republic of Korea; 5 Department of Laboratory Medicine, Hallym University Sacred Heart Hospital, Hallym University College of Medicine, Seoul, Republic of Korea; 6 Department of Laboratory Medicine, Kangnam Sacred Heart Hospital, Hallym University College of Medicine, 1 Shingil-ro, Youngdeungpo-gu, Seoul, 150-950 Korea

**Keywords:** Extended spectrum, Beta-lactamase, Piperacillin-tazobactam, Cefepime, Ertapenem

## Abstract

**Background:**

Due to limited therapeutic options, the spread of extended-spectrum beta-lactamases (ESBLs) have become a major public health concern. We conducted a prospective, randomized, open-label comparison of the therapeutic efficacy of piperacillin-tazobactam (PTZ), cefepime, and ertapenem in febrile nosocomial urinary tract infection with ESBL-producing *Escherichia coli* (ESBL-EC).

**Methods:**

This study was conducted at three university hospitals between January 2013 and August 2015. Hospitalized adult patients presenting with fever were screened for healthcare-associated urinary tract infection (HA-UTI). When ESBL-EC was solely detected and susceptible to a randomized antibiotic in vitro, the case was included in the final analysis. Participants were treated for 10–14 days with PTZ, cefepime, or ertapenem.

**Results:**

A total of 66 participants were evenly assigned to the PTZ and ertapenem treatment groups. After the recruitment of six participants, assignment to the cefepime treatment group was stopped because of an unexpectedly high treatment failure rate. The baseline characteristics of these participants did not differ from participants in other treatment groups. The clinical and microbiological response to PTZ treatment was estimated to be 94% and was similar to the response to ertapenem treatment. The efficacy of cefepime was 33.3%. In the cefepime group, age, Charlson comorbidity index, genotype, and minimal inhibitory concentration (MIC) did not significantly affect the success of treatment. Similarly, genotype seemed to be irrelevant with respect to clinical outcome in the PTZ group. Expired cases tended to involve septic shock with a high Charlson comorbidity index and high MIC.

**Conclusion:**

Results from this study suggest that PTZ is effective in the treatment of urinary tract infection caused by ESBL-EC when the in vitro test indicates susceptibility. In addition, cefepime should not be used as an alternative treatment for urinary tract infection caused by ESBL-EC.

**Trial registration:**

The trial was registered with the Clinical Research Information Service of Korea Centers for Disease Control and Prevention. (KCT0001895)

## Background

The spread of extended-spectrum beta-lactamase (ESBL)-producing organisms has gradually increased in hospitals and long-term care facilities [1]. ESBLs are enzymes that hydrolyze most beta-lactam antibiotics including penicillins, advanced-generation cephalosporins, and aztreonam. The genes of ESBLs are encoded on transferable plasmids, which can carry multiple co-resistance genes for other non-beta-lactam antibiotics [2, 3]. The spread of ESBLs has become a major public health concern due to limited therapeutic options.

Compared to non-ESBL-producing organism infections, those with ESBL-producing organisms are related to poor clinical outcomes [4]. Carbapenems are generally considered the drug of choice for ESBL-producing organism infections due to their stability against ESBLs [5, 6]. However, their use should be restricted considering the emergence of carbapenem-resistant organisms [7]. Alternative treatments are urgently needed to relieve the selective pressure for carbapenem [8, 9]. Thus, over the past few decades, numerous studies have been conducted to determine possible alternatives.

Currently, the most frequently mentioned alternative treatments are beta-lactam/beta-lactamase inhibitors (BLBLI), cephamycins, cefepime, and aminoglycosides [10–20]. Results have been promising, but several studies have reported suboptimal outcomes of cefepime or piperacillin-tazobactam (PTZ) treatment [21–23]. Because previous studies were conducted with observational methods, these conflicting results could be due to confounding factors, such as mixed sources of infection, variability in dosing, and different patient characteristics. To overcome the limitations of observational studies, we conducted a prospective, randomized, open-label comparison of the therapeutic efficacy of PTZ, cefepime, and ertapenem in patients with febrile nosocomial urinary tract infection (UTI) with ESBL-producing *Escherichia coli* (EBSL-EC).

## Methods

### Study setting

This study was conducted at three university hospitals between January 2013 and August 2015. Hospitalized adult patients (≥ 19 years of age) presenting with fever were screened for healthcare-associated UTI (HA-UTI), which was defined according to the CDC/NHSN surveillance recommendations [24]. Exclusion criteria were presence of suspicious or confirmatory infectious foci other than HA-UTI, any use of antibiotics within 7 days prior to recruitment for any reason, any complicating urinary factors that could not be effectively treated during the trial (such as obstruction, suspected or confirmed prostatitis, and epididymitis), indwelling urinary catheters expected to remain in place after completion of therapy, and need for renal replacement therapy. After providing written consent, participants were randomly assigned to receive treatment for 10–14 days with PTZ, cefepime, or ertapenem at each institute, in that order. Clinical data on age, gender, comorbidities, Charlson comorbidity index (CCI), and APACHE II score were collected. On day 5–7 of the initial therapy, the investigator at each institute performed a urine culture to determine whether continuation of the study therapy was appropriate. When ESBL-EC was solely detected and was susceptible to a randomized antibiotic regardless of the sensitivities to other antibiotics, the case was included in the final analysis. If a patient receiving a randomized antibiotic dropped out, that antibiotic was given to the next participant. Because randomization was performed at each institute, a laboratory center monitored the balance in sample sizes across the groups over time. This study was performed in accordance with the CONSORT (Consolidated Standards of Reporting Trials) statement.

### Antibiotic regimen

All patients received doses adjusted according to renal function. For PTZ, patients with creatinine clearance (Ccr) > 40 mL/min were treated with 4.5 g every 6 h, those with Ccr of 20-40 mL/min received 2.25 g every 6 h, and those with Ccr < 20 mL/min received 8 g every 8 h. For cefepime, patients with Ccr > 60 mL/min were treated with 2 g every 12 h, those with Ccr of 30-60 mL/min received 2 g every 24 h, and those with Ccr < 30 mL/min received 1 g every 24 h. For ertapenem, patients with Ccr > 30 mL/min were treated with 1 g every 24 h, and those with Ccr ≤ 30 mL/min received 500 mg daily.

### Bacterial isolates

Urine and blood cultures were conducted in the microbiological laboratory at each hospital prior to antibiotic therapy. To evaluate the microbiological response, urine culture was repeated on day 10–14. At each hospital, microbiological identification was carried out using the Vitek 2 system (bioMérieux Vitek, Hazelwood, MO). Vitek GNI cards containing an ESBL test were used. Susceptibility to multiple antibiotics (including amikacin, ampicillin, ampicillin-sulbactam, aztreonam, cefepime, cefotaxime, cefotetan, ceftazidime, cephalothin, ciprofloxacin, ertapenem, gentamicin, imipenem, PTZ, and trimethoprim-sulfamethoxazole) was recorded. When an ESBL-EC was isolated, the sub-cultured specimen was delivered to Kangnam Sacred Heart Hospital for genotyping of ESBLs, AmpC beta-lactamases, and carbapenemases. For ESBLs-positive isolates, a PCR and sequencing strategy was used to characterize enzymes related to the ESBLs (TEM, SHV, CTX-M, and GES), AmpC beta-lactamases (DHA, MOX, and CMY), and carbapenemases (KPC, NDM, IMP, VIM, and OXA-48) using primers previously described [25–29]. CTX-M type sequencing primers used in this study are summarized in Table 1. Using two primer pairs, we amplified genes included in the CTX-M-1 (*bla*_CTX-M-1_, *bla*_CTX-M-3_, and *bla*_CTX-M-15_) and CTX-M-9 groups (*bla*_CTX-M-9_, *bla*_CTX-M-14_, and *bla*_CTX-M-27_). Then we sequenced the PCR products using identical primer pairs to identify each specific *bla*_CTX-M_ gene. The identified nucleotide sequences were compared with reference *bla*_CTX-M_ alleles (http://www.lahey.org/studies/). We performed species identification using the Vitek 2 system but did not identify the strain using multilocus sequence typing or pulsed field gel electrophoresis.

**Table 1:** Primers used for PCR amplification and sequencing of *bla*_CTX-M_ genes

Target	Name of primer	Sequence (5′ ➔ 3′)	Expected size of amplicon (bp)	Reference
CTX-M-1 group	CTX-M-1F CTX-M-1R	GCAGCACCAGTAAAGTGATGGGCTGGGTGAAGTAAGTGACC	591	[28]
CTX-M-9 group	CTX-M-9F CTX-M-9R	GCTGGAGAAAAGCAGCGGAGGTAAGCTGACGCAACGTCTG	474	[29]

### Clinical and microbiological efficacy

Clinical and microbiological responses were evaluated by the investigators on day 3–5, 10–14, and 28–30. Clinical success was defined as resolution of fever and symptoms of UTI present at entry with no development of new symptoms. If clinical improvement was not achieved until day 3–5, the case was defined as a clinical failure. Microbiological success was defined as elimination of ESBL-producing *E. coli* on a urine culture performed on day 10–14. Emergence of *E. coli* resistance to randomized antibiotic treatment, relapse rate, reinfection rate, and 28-day mortality were evaluated on day 28–30.

### Statistical analysis

One-way analysis of variance (ANOVA) with post-hoc Bonferroni analysis was used to compare continuous variables among the three groups. Chi-square and Fisher’s exact tests were used for bivariate analyses. To identify risk factors for treatment failure, multivariate analysis is generally used. However, there were too few failure cases to conduct this analysis. Therefore, a descriptive approach was used in the genotype and MIC analyses. All *p*-values were two sided and accepted when *p* < 0.05. Statistical analysis was performed using SPSS 18.0 software (SPSS Korea, Seoul, Korea).

## Results

### Study subjects

During the study period, a total of 72 participants were enrolled. Of these, 66 participants were evenly assigned to the PTZ and ertapenem treatment groups. After recruitment of six participants to the cefepime treatment group, allocation to this treatment group was stopped due to an unexpectedly high treatment failure rate. Table 2 shows the baseline characteristics of the participants. The average age of participants (65 years) did not vary among the three groups. There were more female than male participants assigned to both the PTZ (female 90.9%) and ertapenem (female 78.8%) treatment groups, but significant gender differences were not observed between the two groups (*p* = 0.303). In the cefepime group, there was an equal distribution of female and male participants, and the gender ratio was significantly different from the two other groups (*p* = 0.049). With respect to comorbidities, the Charlson comorbidity index was similar among the three groups. Almost 65% of the participants had at least one or more underlying disease. Septic shock and concomitant bacteremia were presented in 20–30% of participants in the PTZ and ertapenem groups and did not show statistical differences. APACHE II scores were similar among the three groups. Septic shock and bacteremia were not detected in the cefepime group.

**Table 2:** Demographic characteristics of study subjects

	Piperacillin/tazobactam (*N* = 33)	Cefepime (*N* = 6)	Ertapenem (*N* = 33)	*p*-value
Age	68.8 ± 14.4	75.3 ± 6.6	65.2 ± 16.9	0.281
Female	30 (90.9)	3 (50.0)	26 (78.8)	0.049
Comorbidity, n (%)				
Ischemic heart disease	0 (0)	0 (0)	1 (3.0)	1.000
Diabetes mellitus	12 (36.4)	1 (16.7)	15 (45.5)	0.474
Cerebrovascular accident	5 (15.2)	1 (16.7)	2 (6.1)	0.420
Dementia	3 (9.1)	0 (0)	2 (6.1)	1.000
Hemiplegia	2 (6.1)	0 (0)	2 (6.1)	1.000
Congestive heart failure	5 (15.2)	1 (16.7)	1 (3.0)	0.230
COPD	1 (3.0)	0 (0)	1 (3.0)	1.000
Chronic kidney disease	2 (6.1)	0 (0)	2 (6.1)	1.000
Liver cirrhosis	2 (6.1)	0 (0)	4 (12.1)	0.809
Solid tumor	6 (18.2)	1 (16.7)	7 (21.2)	1.000
Lymphoma	1 (3.0)	0 (0)	2 (6.1)	1.000
None	12 (36.4)	2 (33.3)	12 (36.4)	1.000
Charlson comorbidity index	4.7 ± 3.0	4.7 ± 1.0	4.5 ± 3.0	0.951
Bacteremia, n (%)	9 (27.3)	0 (0)	7 (21.2)	0.477
Septic shock, n (%)	9 (24.2)	2 (33.3)	11 (33.3)	0.928
APACH II score	12.9 ± 2.9	16.5 ± 6.4	16.6 ± 5.6	0.298

### Clinical and microbiological outcomes

Clinical and microbiological outcomes are summarized in Table 3. Clinical success rate was 93.9% (31/33) with PTZ and 97.0% (32/33) with ertapenem; the rates were not statistically different (*p* = 0.500). However, the clinical success rate with cefepime was 33.3% (2/6), which was significantly lower than those of the other antibiotic groups (*p* < 0.001). The microbiological success rates of PTZ and ertapenem were the same at 97.0% (32/33), while the cefepime group achieved a 33.3% success rate (2/6). The 28-day mortality was also the same between the PTZ and ertapenem groups with a rate of 6.1% (2/33) in both groups. On the other hand, the rate was 33.3% (2/6) in the cefepime group (*p* = 0.108). There were no cases of emergence of *E. coli* resistance to randomized antibiotics, relapse, or reinfection. In the case of microbiological failure, the MICs of late cultures at 10–14 days were not different from early cultures. All patients with a positive culture at test of cure had clinical symptoms that were consistent with UTI.

**Table 3:** Clinical and microbiological outcomes according to the antibiotic groups

	Piperacillin/tazobactam (*N* = 33)	Cefepime (*N* = 6)	Ertapenem (*N* = 33)	*p*-value
Clinical success, n (%)	31 (93.9)	2 (33.3)	32 (97.0)	<0.001
Microbiological success, n (%)	32 (97.0)	2 (33.3)	32 (97.0)	<0.001
Clinical and microbiological success, n (%)	31 (93.9)	2 (33.3)	32 (97.0)	<0.001
28-days mortality, n (%)	2 (6.1)	2 (33.3)	2 (6.1)	0.108

### Genotypic analysis in the cefepime and piperacillin-tazobactam groups

There were no ESBL-EC isolates combined with AmpC or carbapenemase enzymes in this study. In the cefepime group, only two participants achieved clinically successful recovery (Table 4). There were four failure cases and two deaths. While the MIC of cefepime was 1 μg/mL or 2 μg/mL, the successful cases all had an MIC of 2 μg/mL. The genotype was predominantly CTX-M-9, but one case was detected as SHV-2. The genotype did not appear to significantly affect the success of treatment. In addition, age and Charlson comorbidity index did not seem to be directly related to clinical success. All mortality cases occurred under conditions of septic shock.

**Table 4:** Schematic description of clinical outcomes according to MIC, genotype, age, Charlson comorbidity index (CCI), presence of concomitant bacteremia and septic shock in cefepime, piperacillin/tazobactam and ertapenem groups

Case	MIC (μg/mL)	ESBLs genotype	CCI	Bacteremia	Septic shock	Clinical outcome
A. Cefepime (*N* = 6)						
Patient 1	2	CTX-M-14	5	No	No	Success
Patient 2	2	CTX-M-14	3	No	No	Success
Patient 3	1	CTX-M-14	4	No	No	Failure
Patient 4	2	CTX-M-14	6	No	No	Failure
Patient 5	1	SHV-12	5	No	Yes	Failure and expired
Patient 6	2	CTX-M-14	5	No	Yes	Failure and expired
B. Piperacillin/tazobactam (*N* = 33)						
Patient 1	4	CTX-M-14	6	No	No	Success
Patient 2	4	CTX-M-15	5	No	Yes	Success
Patient 3	4	CTX-M-15	0	No	No	Success
Patient 4	4	CTX-M-15	1	No	No	Success
Patient 5	4	CTX-M-27	9	No	No	Success
Patient 6	4	CTX-M-27	9	No	No	Success
Patient 7	4	CTX-M-27	9	No	No	Success
Patient 8	8	CTX-M-14	3	Yes	Yes	Success
Patient 9	8	CTX-M-14	1	No	No	Success
Patient 10	16	CTX-M-1	4	No	No	Success
Patient 11	16	CTX-M-3	2	No	Yes	Success
Patient 12	16	CTX-M-14	3	No	No	Success
Patient 13	16	CTX-M-14	3	Yes	Yes	Success
Patient 14	16	CTX-M-15	1	No	Yes	Success
Patient 15	16	CTX-M-15	4	No	No	Success
Patient 16	16	CTX-M-27	0	No	No	Success
Patient 17	16	CTX-M-15	3	No	No	Success
Patient 18	16	CTX-M-14	5	No	No	Success
Patient 19	16	CTX-M-14	7	No	No	Success
Patient 20	16	CTX-M-14	1	Yes	No	Success
Patient 21	16	CTX-M-14	8	No	No	Success
Patient 22	16	Not tested	5	No	No	Success
Patient 23	16	Not tested	2	No	Yes	Success
Patient 24	16	Not tested	7	No	No	Success
Patient 25	16	Not tested	3	No	No	Success
Patient 26	16	Not tested	7	No	No	Success
Patient 27	16	Not tested	8	No	No	Success
Patient 28	16	Not tested	5	No	No	Success
Patient 29	16	Not tested	5	No	No	Success
Patient 30	16	Not tested	3	Yes	No	Success
Patient 31	16	Not tested	7	Yes	Yes	Success
Patient 32	16	CTX-M-15	9	Yes	Yes	Failure and expired
Patient 33	16	CTX-M-27	10	No	Yes	Failure and expired
C. Ertapenem (*N* = 33)						
Patient 1	0.5	CTX-M-15	0	No	No	Success
Patient 2	0.5	CTX-M-27	0	No	No	Success
Patient 3	0.5	CTX-M-14	1	No	No	Success
Patient 4	0.5	CTX-M-15	1	No	No	Success
Patient 5	0.5	CTX-M-14	1	Yes	No	Success
Patient 6	0.5	CTX-M-15	1	No	Yes	Success
Patient 7	0.5	CTX-M-3	2	No	Yes	Success
Patient 8	0.5	CTX-M-14	2	Yes	Yes	Success
Patient 9	0.5	CTX-M-14	3	No	No	Success
Patient 10	0.5	CTX-M-15	3	No	No	Success
Patient 11	0.5	CTX-M-15	3	No	No	Success
Patient 12	0.5	CTX-M-14	3	Yes	No	Success
Patient 13	0.5	CTX-M-14	3	Yes	Yes	Success
Patient 14	0.5	CTX-M-14	3	Yes	Yes	Success
Patient 15	0.5	CTX-M-1	4	No	No	Success
Patient 16	0.5	CTX-M-15	4	No	No	Success
Patient 17	0.5	CTX-M-14	5	No	No	Success
Patient 18	0.5	CTX-M-14	5	No	No	Success
Patient 19	0.5	CTX-M-15	5	No	No	Success
Patient 20	0.5	CTX-M-14	5	Yes	No	Success
Patient 23	0.5	CTX-M-15	5	No	Yes	Success
Patient 21	0.5	CTX-M-14	6	Yes	No	Success
Patient 22	0.5	CTX-M-14	7	No	No	Success
Patient 24	0.5	CTX-M-14	7	No	No	Success
Patient 25	0.5	CTX-M-14	7	No	No	Success
Patient 26	0.5	CTX-M-14	8	No	No	Success
Patient 27	0.5	CTX-M-14	8	No	No	Success
Patient 28	0.5	CTX-M-27	9	No	No	Success
Patient 29	0.5	CTX-M-27	9	No	No	Success
Patient 30	0.5	CTX-M-27	9	No	No	Success
Patient 31	0.5	CTX-M-27	10	No	No	Success
Patient 32	0.5	CTX-M-14	9	Yes	Yes	Failure and expired
Patient 33	0.5	CTX-M-15	7	Yes	Yes	Failure and expired

Not tested: The isolate was ESBLs-positive by Vitek-2 system but not tested the ESBLs genotyping due to loss of the isolate

In the PTZ group, treatment was successful except in two cases (Table 4). In most cases, the MIC was 16 μg/mL and accounted for 72.7% of the total. Although the clinical outcome was satisfactory in most cases of 16 μg/mL MIC, all failure and mortality cases were in the 16 μg/mL MIC group. Ten samples were lost during transport or over the course of the experiment. CTX-M-14, CTX-M-15, and CTX-M-27 were frequently observed. The genotypes of the mortality cases were CTX-M-15 or CTX-M-27. Similar to cefepime, the genotype seemed to be irrelevant with respect to clinical outcome. Deaths tended to be associated with septic shock with high Charlson comorbidity index and high MIC.

## Discussion

This is the first randomized study comparing the efficacy of PTZ, cefepime, and ertapenem. Although the sample size was small, results from the study showed that PTZ was as effective as ertapenem for the treatment of ESBL-EC UTI. Clinical and microbiological response to PTZ treatment was estimated to be 94%. Unexpectedly, the efficacy of cefepime was only 33.3%, suggesting that cefepime is not an appropriate therapeutic alternative for ESBL-EC UTI.

ESBLs might be inhibited by beta-lactamase inhibitors; thus, it is theoretically attractive to use BLBLI combinations to treat ESBL infections. In fact, a large, multicenter, prospective observational study has reported that outcomes using BLBLIs were comparable to those with carbapenem in the treatment of ESBL-EC blood stream infection [10]. In addition, a recent meta-analysis found no statistical differences in mortality between carbapenem treatment and BLBLI treatment in patients with bacteremia caused by ESBL-producing pathogens [30]. However, in another study, BLBLI appeared to be inferior to carbapenem for treatment of bacteremia [31]. These inconclusive results might be due to differences in the proportion of bacteremia sources among the various studies since the infection site can significantly influence the therapeutic efficacy of antibiotics. To overcome issues due to infection heterogeneity, this study focused on the treatment of UTIs.

According to our results, PTZ is a reliable alternative in the treatment of ESBL-EC-proven UTI. An inoculum effect has been proposed as a major limitation of PTZ [32]. PTZ has some merits for use in cases of UTI. Tazobactam is mainly excreted in the urine, and its high concentration in the urine is noted in the presence of piperacillin [33]. In addition, UTIs can have a relatively lower bacterial burden than other infectious diseases, such as pneumonia, complicated intra-abdominal infection, and blood stream infection. Therefore, PTZ might be able to overcome the inoculum effect in UTIs. Interestingly, mortality cases were found in participants with a high MIC who received PTZ treatment. Due to the small sample size, it was difficult to determine whether a higher MIC of PTZ is an important risk factor for treatment failure. However, in this study, multiple cases with a 16 μg/mL MIC of PTZ were successfully treated. As discussed in a previous study, the MIC might not be a significant risk factor in UTIs [16]. Treatment failure seems to be closely related to the patient’s baseline conditions, irrespective of the MIC.

Cefepime is frequently used for treatment of health-care associated infections and shows greater stability in vitro against ESBL-producing pathogens than other cephalosporins [34]. Some clinical studies have reported successful treatment using cefepime in cases of ESBL-producing bacterial infection [19, 35]. However, several other studies have shown disappointing outcomes when using cefepime to treat bacteremic conditions [20, 23]. Cefepime is highly vulnerable to the inoculum effect, and a high MIC is an important risk factor for treatment failure [32]. As seen in our study, cefepime was not effective in the treatment of UTIs even in non-bacteremic conditions. Treatment failure was also observed despite an MIC of 1 μg/mL or 2 μg/mL. Thus, a lower MIC does not predict clinical success in cefepime treatment. Although cefepime is excreted mostly unchanged in urine, it can be easily inactivated by ESBLs in UTIs. Otherwise, the results we observed might be due to the emergence of phenotypic heterogeneous resistance to cefepime during treatment [36]. Another cause of treatment failure could be under-dosing of cefepime. In Korea, cefepime has been approved to be administered at 1 g twice a day for mild or moderate infection, 2 g twice a day for severe infection, and 2 g three times a day for neutropenic patients if renal function is normal. The recommended dose is the same in most other countries. However, some studies recommended higher doses of cefepime than usual for clinical doses. One study reported that doses of at least 2 g every 8 h are required to treat infections considering clinical pharmacodynamics [37]. However, that study enrolled patients with non-urinary tract infections, and the pathogen of focus was *Pseudomonas aeruginosa*. Therefore, it is difficult to infer the same conclusion from this study. Other studies using a series of 5000-subject Monte Carlo simulations mentioned that a cefepime dose of 2 g every 6 h provided favorable probability [38]. Considering results from existing studies, further clinical studies increasing the dose of cefepime seem to be necessary to clarify the failure of cefepime.

This study has several limitations. First, the statistical power was low due to the small number of participants. To estimate the sample size for clinical research studies, the variance or standard deviation is obtained from previous studies. When there are no previous studies, a formal sample size calculation might not be appropriate. We decided to complete the study according to the study period regardless of the sample size, as in the pilot study. During the study period, the number of patients susceptible in vitro to PTZ was unexpectedly small. Furthermore, the exclusion criteria were strict in order to reduce possible confounding factors. Accordingly, the sample size was only 33 participants in each group except the cefepime group; however, this is a common pilot sample study size for a two-arm trial [39]. In order to have more confidence in the outcome, a larger sample size is needed in future studies. Second, it has been suggested that ESBL-*Klebsiella pneumoniae* is associated with higher mortality than ESBL-EC bacteremia [40]. Therefore, the results could not be generalized to pathogens other than *E. coli*. Third, the genotype was not determined in some cases due to loss of the isolate. Fourth, the molecular PCR typing was not done for cefepime resistance gene such as OXA-30. Results could be interpreted differently in situations with other ESBL genotypes. In the Republic of Korea, the predominant types of ESBLs in *E. coli* are CTX-M-14 and CTX-M-15, which is consistent with the results of the tested isolates in our study [41]. In our study, the tested isolates demonstrated similar predominance. Therefore, these results could be applied to the situation of high spread of the CTX-M type.

## Conclusion

Alternatives for the treatment of ESBL-producing bacteria are urgently needed to suppress the emergence of carbapenem-resistant pathogens. Results from this study suggest that PTZ is effective in the treatment of UTI caused by ESBL-EC when the in vitro test indicates susceptibility. Empirical PTZ therapy for healthcare-associated UTI seems to be reasonable if the hospital epidemiological antimicrobial pattern of ESBLs (especially the CTX-M type) is dominantly in vitro susceptible to PTZ. In addition, cefepime should not be used as an alternative treatment in urinary tract infections caused by ESBL-EC.

## Abbreviations

ESBL-EC: ESBL-producing *Escherichia coli*
ESBLs: Extended-spectrum beta-lactamases
HA-UTI: Healthcare-associated urinary tract infection
MIC: Minimal inhibitory concentration
PTZ: Piperacillin-tazobactam
UTI: Urinary tract infection
